# The absence of IL-9 reduces allergic airway inflammation by reducing ILC2, Th2 and mast cells in murine model of asthma

**DOI:** 10.1186/s12890-022-01976-2

**Published:** 2022-05-06

**Authors:** Yan Li, Feng Lan, Yiran Yang, Yingjie Xu, Yalin Chen, Xiaofeng Qin, Zhe Lv, Wei Wang, Sun Ying, Luo Zhang

**Affiliations:** 1 Department of Otorhinolaryngology Head and Neck Surgery, Beijing Tongren Hospital, Capital Medical University, Beijing Institute of Otorhinolaryngology, Key Laboratory of Otorhinolaryngology Head and Neck Surgery, Ministry of Education, Beijing Key Laboratory of Nasal Diseases, No. 17, HouGouHuTong, DongCheng District, Beijing, 100730 China; 2 Department of Immunology, School of Basic Medical Sciences, Capital Medical University, 10 Xi Tou Tiao, You An Men Wai, Fengtai District, Beijing, 100069 China; 3 Department of Thyroid Head and Neck Surgery, Qingdao Central Hospital, Qingdao University, Qingdao, China

**Keywords:** Asthma, IL-9, ILC2, Th2, Mast cell, Airway inflammation

## Abstract

Allergic asthma is an allergic inflammatory disease of the airways, in which numerous cell types and cytokines have been shown to contribute to pathogenesis of the disease. Although increased expression of IL-9 has been shown to influence the activity of structural as well as eosinophils and mast cells in asthma, the influence of IL-9 on function of ILC2 and Th2 cells remains unclear. This study therefore aimed to elucidate the role of IL-9 on ILC2 and Th2 cells using a murine model of asthma. A murine model of asthma was established using wild type (WT) and IL-9-deficient (*Il9*^*−/−*^*)* transgenic mice sensitized to house dust mite (HDM). Bronchoalveolar lavage fluid (BALF) and lung tissues were collected, and analysed for inflammatory cells (eosinophils, mast cells, Th2 cells and ILC2 cells), histopathological changes, and several cytokines. HDM challenge significantly increased accumulation of ILC2 cells, Th2 cells and mast cells, as well as goblet cell hyperplasia, and the expression of cytokines IL-4, IL-5 and IL-13, but not IFN-γ, in WT mice compared to saline-challenged control group. In contrast, all pathological changes, including infiltration of ILC2 cells, Th2 cells and mast cells, were significantly attenuated in HDM-challenged *Il9*^*−/−*^ mice. Furthermore, the number of Ki67^+^ILC2 cells, Ki67^+^Th2 cells and Ki67^+^mast cells were significantly reduced in the absence of IL-9 signalling. These data suggest that IL-9 promotes the proliferation and type 2 cytokine production of type 2 cells in the murine models of asthma, and therefore might be a potential therapeutic target for asthma treatment.

## Introduction

Asthma is a common airway inflammatory disease involving cells of both the innate and adaptive immune systems. Currently, approximately 8% of people in developed countries have asthma compared with 4.3% of the population worldwide, and both numbers are on the rise [1]. Various inflammatory cell types, including T cell, B cell, ILC2 cell, eosinophil, and mast cell, are involved in the complex allergic inflammatory response in the airways [2]. In particular, Th2 cells and ILC2 cells are thought to play major roles in initiation and maintenance of the allergic inflammation [2]. Production of cytokines such as IL-4, IL-5, IL-9, and IL-13 by these cells is thought to be involved in the development of asthmatic features, including pulmonary eosinophilia, mucus overproduction, mast cell hyperplasia, and bronchial hyperresponsiveness. However, the precise roles of the triggers and mediators involved in generating these processes remain to be clarified.

IL-9, a pleiotropic cytokine, can promote allergic inflammation and is associated with various pathophysiological changes observed in the airways of asthmatic patients, such as eosinophilic inflammation, mucous gland hyperplasia and hypertrophy, and bronchial hyperresponsiveness [3]. Consistent with elevated expression of IL-9 in peripheral blood mononuclear cells and lung tissues of asthmatic patients [4, 5], over-expression of IL-9 results in accumulation of eosinophils, mucus overproduction, and mast cell hyperplasia [3, 6, 7]. Furthermore, anti-IL-9 treatment ameliorates airway inflammation in mouse models of asthma employing ovalbumin sensitization and challenge [8–11]. Moreover, IL-9 promotes mast cell growth in vitro [12], while neutralization of IL-9 markedly diminished mucosal mast cell activation and mast cell numbers in the lung in a mouse model of asthma [10]. Despite the evidence for the effect of IL-9 on mast cells in asthma, whether IL-9 affects the other effector cells in asthma remains unclear.

Although IL-9 was originally described as a T cell growth factor in vitro [13], its role on T lymphocyte responses presently remains controversial. Treatment with an anti-IL-9 antibody markedly reduces the number of Th2 cells in a mouse of allergic rhinitis [14], however, anti-IL-9 treatment does not affect ovalbumin-induced Th2 cytokine production by mice spleen T cells in vitro [11]. Thus, it is worthy to investigate the mode of action of IL-9 and whether this cytokine acts on ILC2 and Th2 cells in allergic airway inflammation.

In the present study, we have established a murine model of asthma using wild type (WT) and IL-9-deficient (*Il9*^*−/−*^) mice and then investigated the distribution of mast cells, Th2 cells and ILC2 cells, following sensitization to and challenge with HDM. Furthermore, the potential role of IL-9 in regulation of these effector cells and allergic inflammation was also investigated.

## Materials and methods

### Animals

Female BALB/c mice (8–10 weeks old; Vital River Laboratory, Beijing, China) were kept in a pathogen-free environment in the Department of Laboratory Animal Sciences, Capital Medical University, Beijing, China. *Il9*^*−/−*^mice (BALB/c background) were kindly provided by Professor Andrew McKenzie (Medical Research Council Laboratory of Molecular Biology, Cambridge, UK). All mice were randomly divided into different groups (10 mice per group). All animal studies were carried out strictly under protocols approved by the Institutional Animal Care and Use Committee at Capital Medical University, Beijing, China.

### Murine models

**Fig. 1:**
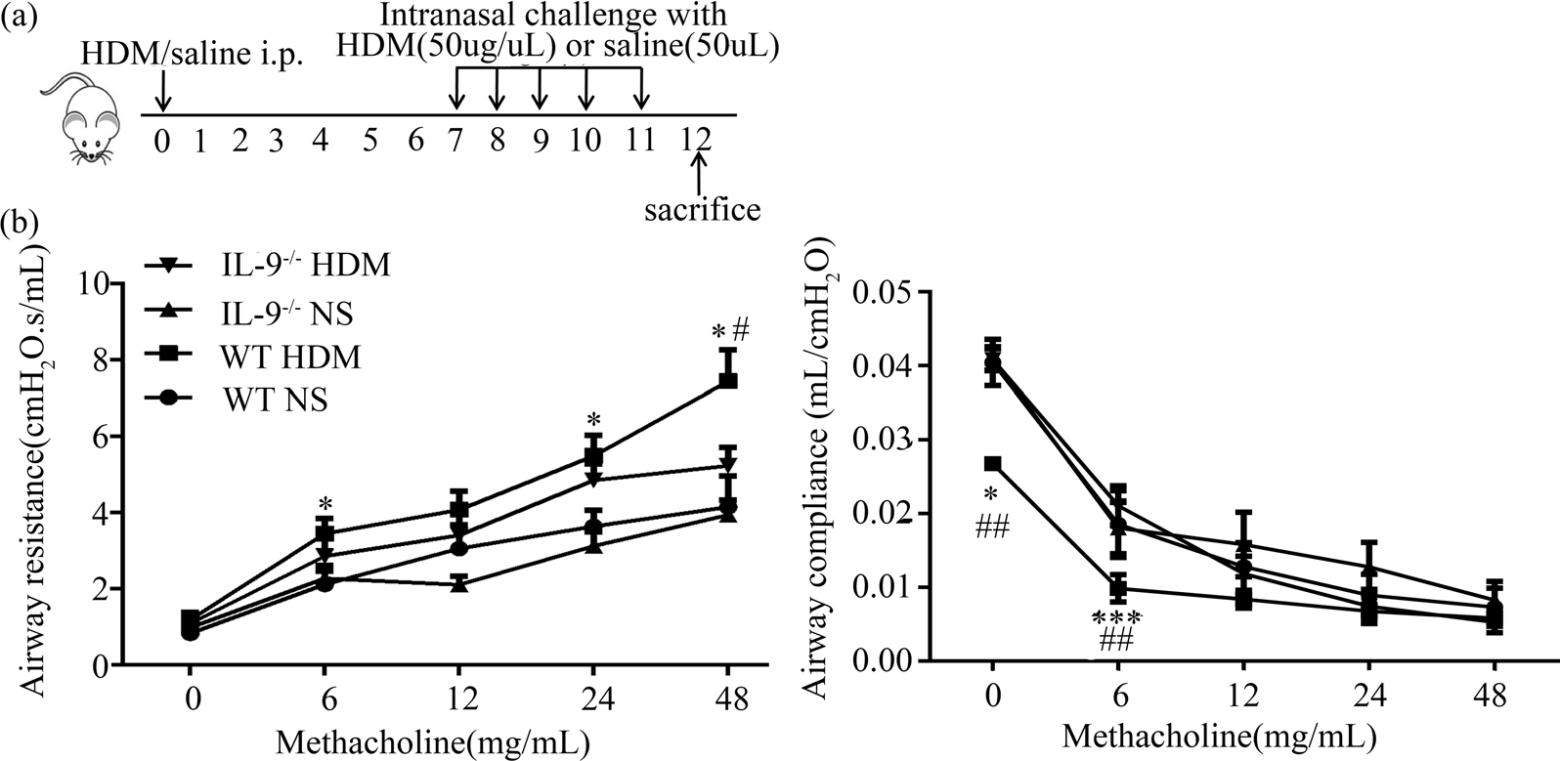
IL-9 deficiency decreased HDM-induced airways resistance. **a** Schedule of allergen challenge in mice. **b** Airways resistance (Rrs) and compliance (Crs) of wild type (WT) and IL-9 deficient (*Il9*^*−/−*^) mice challenged with HDM or saline (NS) control. *p < 0.05, ***p < 0.001 versus NS challenged wild type mice, ^#^*p* < 0.05, ^##^*p* < 0.01 versus *Il9*^*−/−*^ mice challenged with HDM. Data are presented as the mean ± SEM (n = 5 in each group)

Murine surrogates of asthma induced by HDM challenge were prepared and studied as previously described [15]. Briefly, WT and *Il9*^*−/−*^ mice were randomly assigned to two groups. The mice in the HDM-challenged group were first sensitized (day 0) by intraperitoneal injection of 100 μg HDM (Cosmo Bio Co LTD), emulsified in AL[OH]_3_/dose. Seven days later, the mice were challenged daily for five consecutive days with 50 μg of HDM in 50 μL saline/dose by intranasal instillation. Mice in the saline control challenge group were injected intraperitoneally with the same amount of AL[OH]_3_ and intra-nasally with saline at the same time points corresponding to those in the HDM challenge group (Fig. 1a). The mice were sacrificed 24 h after the final challenge and bronchoalveolar lavage fluid (BALF) and lung tissues were collected from each animal.

### Lung function measurement

Twenty-four hours after the last intranasal HDM challenge, airway responsiveness was assessed on day 12 using the FlexiVent system (SCIREQ Inc., Montreal, QC, Canada) following the manufacturer’s protocol, as previously described [16, 17]. Briefly, after the anesthetization with 80 mg/kg pentobarbital sodium (Sigma-Aldrich, St. Louis, MO, USA) by intraperitoneal injection, mice were conducted with tracheotomy and endotracheal intubation, And then airway responsiveness was assessed by measuring the changes of lung resistance after sequentially exposed to increasing doses of methacholine (Mch, Sigma-Aldrich) in sterile saline (0, 6, 12, 24 and 48 mg/mL). The values of airways resistance (Rrs) and compliance (Crs) were recorded for analyzing the airways hyperresponsiveness (AHR).

### Collection of BALF and characterization of cellular infiltrate

BALF was collected from the mice and total cells were enumerated as previously described [15]. Cellular smears were then prepared and stained with haematoxylin& eosin (H&E) for differential cellular counts [16].

### Lung histology

Left lung tissue from each animal was fixed in 4% paraformaldehyde and embedded in paraffin wax prior to cutting into Sects. (5 μm thickness). The sections were stained with H&E for scoring of inflammatory cellular infiltration under light microscopy by operators ignorant of the provenance of the sections, as previously described [16, 17]. Briefly, the numbers of peribronchial, infiltrating cells were scored globally visually as 0–5, where 0 = no cells; 1 = a few cells; 2 = at least one layer of peribronchial cells; 3 = 2–4 layers of peribronchial cells; 4 =  > 4 layers of peribronchial cells; 5 = abundant proximal and distal cellular infiltration. 6–10 airways were counted per mouse.

Additional sections were stained with periodic acid-Schiff (PAS) for detection of mucus deposition. PAS staining was assessed semi-quantitatively in sections of central and peripheral airways as the mean score of typically 14 central and peripheral airways, where 0 =  ≤ 5% goblet cells; 1 = 5–25%; 2 = 25–50%; 3 = 50–75%; 4 =  ≥ 75%. Further lung sections were stained with Congo red stain for detection of eosinophil infiltration [16, 17]. Digital photographs of 10 bronchioles per tissue section were taken at 40× magnification and Image-Pro Plus was used to objectively quantify Congo red stained eosinophils, as previously described [16, 17].

### Cytokines analysis

Resected right lungs were weighed then homogenised in PBS containing 1% Triton X-100 and protease inhibitor cocktail (RocheDiagnostics GmbH, Mannheim, Germany). After centrifugation to remove debris, the supernatants were collected for measurement of cytokines. The concentrations of IL-4, IL-5, IL-6, IL-13, IL-17A, IL-25, IL-33, IFN-γ, mast cell protease 1 (mMCP-1) and thymic stromal lymphopoietin (TSLP) were measured using commercial ELISA kits (Invitrogen, San Diego, CA). The concentrations of serum total IgE antibody was measured by ELISA (Invitrogen). We have measured the expression of HDM specific IgE and IgG_1_ in the sera [16, 17]. Briefly, 96-well microtiter plates were coated with 100μL volume of 10 μg/mL of a whole protein of HDM overnight. Serum samples were diluted 1:10 (for IgE) or 1:1000 (for IgG_1_), and antibody detected using goat anti-mouse IgE (HRP) (1:5000, Invitrogen) or goat anti-mouse IgG_1_ (HRP) (1:5000, Abcam, Cambridge, MA) at 37 °C for 1 h.

### Preparation of single-cell suspensions and flow cytometric analysis

To compare cellular profiles after the induction of airway inflammation, single-cell suspensions were prepared from lungs and BALF cells. Lungs were cut into pieces and digested in DPBS medium containing DNase I (Sigma-Aldrich) (50 U/mL) and collagenase VIII (Sigma-Aldrich) (250 U/mL) at 37 °C for 30 min. Single-cell suspensions were obtained by passing the lung tissue digest through a 70-μm cell strainer. The cell suspensions were stained for different cell types using specific antibodies, prior to assessment by flow cytometry as follows: APC-anti-mouse Lineage cocktail (BD pharmingen, San Jose, CA), APC-Cy7-anti-mouse CD45 (30-F11, BD pharmingen), BV510-anti-mouse ST2 (U29-93, BD pharmingen), PE-anti-mouse ICOS (7E.17G9, BD pharmingen), BV650-anti-mouse CD4 (RM4.5, BD pharmingen), BUV395-anti-mouse GATA3 (L50-823, BD pharmingen), BV711-anti-mouse CD117 (2B8, BD pharmingen), Percp-Cy5.5-anti-mouse FcεRIα (MAR-1, Biolegend, San Diego, CA), PE-Cy7-anti-mouse Ki67 (SolA15, eBioscience, Santa Clara, CA). Isotype and single-stain controls were included. The samples were processed using the LSRFortessa (BD Biosciences, San Jose, CA) flow cytometer and evaluated with Flow Jo software (version V10, Tree Star, Inc., Ashland OR).

### Statistical analysis

Data were expressed as the mean ± SEM and the unpaired t-test was used to analyse the significance of any differences between the groups. All statistical analyses were performed using Graph Pad Prism software and a value of *P* < 0.05 was considered to be statistically significant.

## Results

### IL-9 deficiency attenuated HDM-induced airway hypersponsiveness

WT and *Il9*^*−/−*^ mice were intranasally challenged with HDM or saline control as described in the Methods (Fig. 1a). Consistent with our previous report [15], HDM challenge induced a significant elevation of airways resistance (Rrs), and a markedly reduce of the compliance (Crs) values compared with saline challenge in WT mice. However, the phenomena were significantly reversed in *Il9*^*−/−*^ mice compared to WT mice following HDM challenge (Fig. 1b). And there was no significant difference between HDM-challenged *Il9*^*−/−*^ mice and saline-challenged *Il9*^*−/−*^ mice in terms of Rrs and Crs (Fig. 1b).

### IL-9 deficiency attenuated HDM-induced inflammatory cellular infiltration of the lung parenchyma tissues and airways

**Fig. 2:**
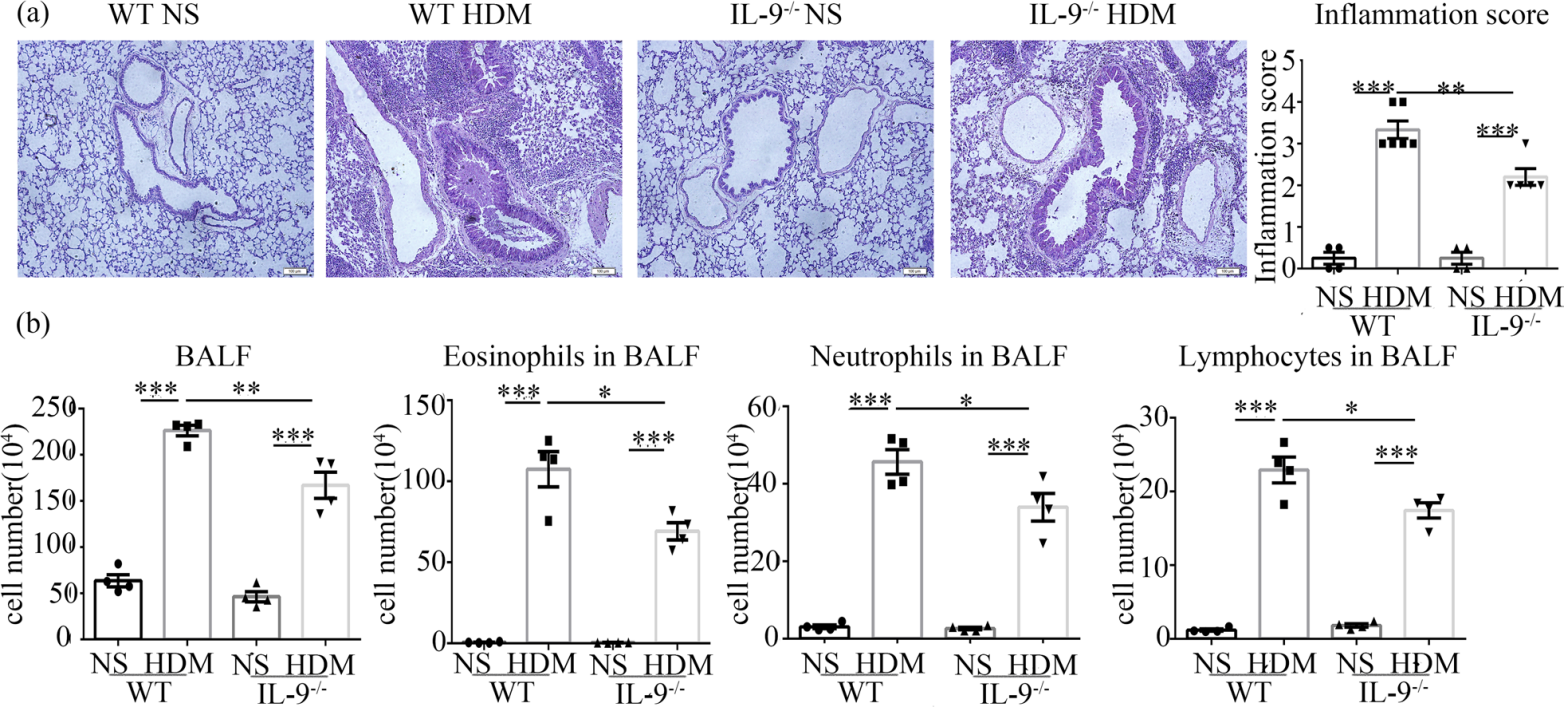
IL-9 deficiency decreased HDM-induced inflammatory cellular infiltration. **a** Left: representative photomicrographs of haematoxylin and eosin-stained lung sections from WT and *Il9*^*−/−*^mice challenged with HDM and saline (magnification ×10). Right: semi-quantitative scoring of the severity of inflammatory infiltrates in the airways. **b** Numbers of total cells, eosinophils, neutrophils and lymphocytes in bronchoalveolar lavage fluid (BALF) of WT and *Il9*^*−/−*^mice challenged with HDM and NS. Bars show the mean ± SEM (n = 4–6 in each group). *p < 0.05, **p < 0.01, ***p < 0.001

Intranasal challenge with HDM resulted in marked infiltration of inflammatory cells into the airway lumen and peribronchial and perivascular regions of the lung tissues, compared with saline control (Fig. 2a, b). Most of the cells infiltrating the lumen were eosinophils, neutrophils and lymphocytes with eosinophils predominating (Fig. 2b). Although HDM challenge also induced inflammatory cellular infiltration into the lungs in the *Il9*^*−/−*^ mice, the numbers of infiltrating inflammatory cells were significantly lower in the *Il9*^*−/−*^ mice compared with that in the WT mice (Fig. 2b).

### IL-9 deficiency decreased HDM-induced eosinophil infiltration into the airways

**Fig. 3:**
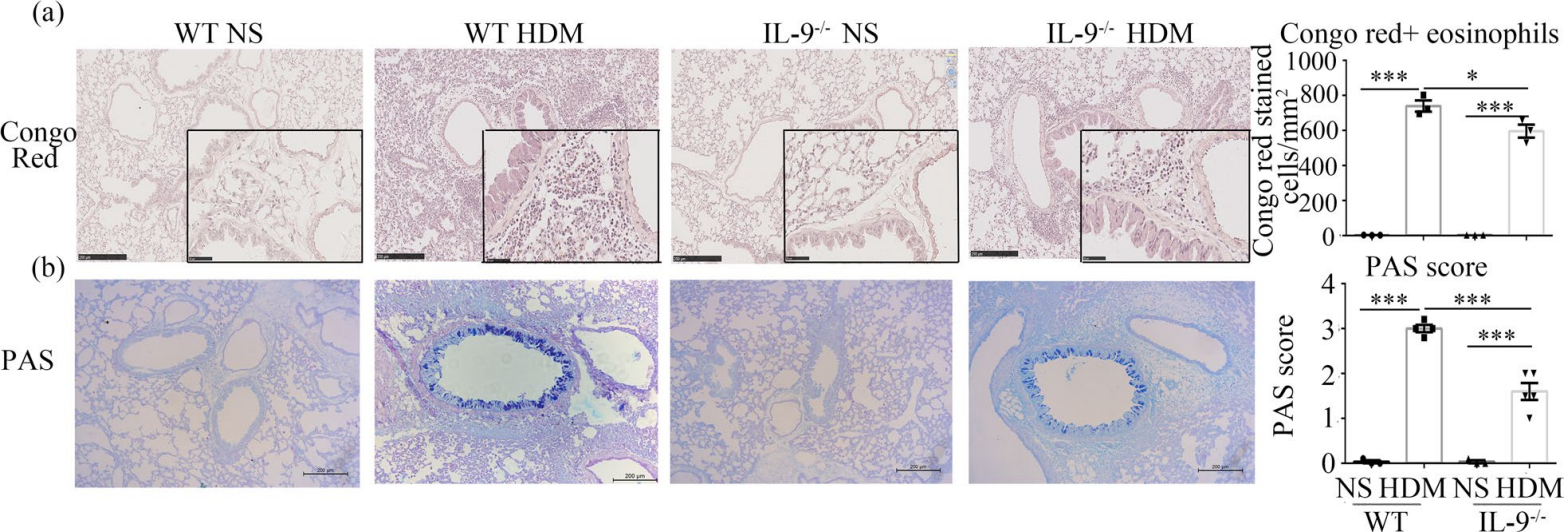
IL-9 deficiency decreased HDM-induced eosinophil infiltration and goblet cell hyperplasia. **a** Left: representative photomicrographs of Congo red-stained sections of lung tissues from WT and *Il9*^*−/−*^mice challenged with HDM and NS (magnification ×10 and × 40). Right: numbers of Congo red^+^ cells per unit area (mm^2^). **b** Left: representative photomicrographs of PAS-stained sections of lung tissue from WT and *Il9*^*−/−*^ mice challenged with HDM and NS (original magnification ×10). Right: mucus score based on PAS staining (see “Materials and methods”). Bars show the mean ± SEM (n = 3–5 in each group). *p < 0.05, **p < 0.01, ***p < 0.001

Congo red staining confirmed the presence of abundant eosinophils in the airway peribronchial and perivascular regions of the WT mice challenged with HDM compared with control mice challenged with saline (Fig. 3a), and that eosinophilic infiltration in the HDM-challenged *Il9*^*−/−*^ mice was significantly attenuated compared with HDM-challenged WT mice (Fig. 3a).

### IL-9 deficiency decreased HDM-induced airway goblet cell hyperplasia

PAS staining demonstrated that HDM inhalation challenge also significantly increased mucous hyperplasia in the bronchial epithelium of WT mice compared to control saline challenge, and that IL-9 deficiency attenuated the HDM-induced goblet cell hyperplasia, as indicated by significantly lower mean PAS score in the *Il9*^*−/−*^ mice compared to WT animals (Fig. 3b).

### IL-9 deficiency attenuated HDM-induced production of cytokines in lung homogenates

**Fig. 4:**
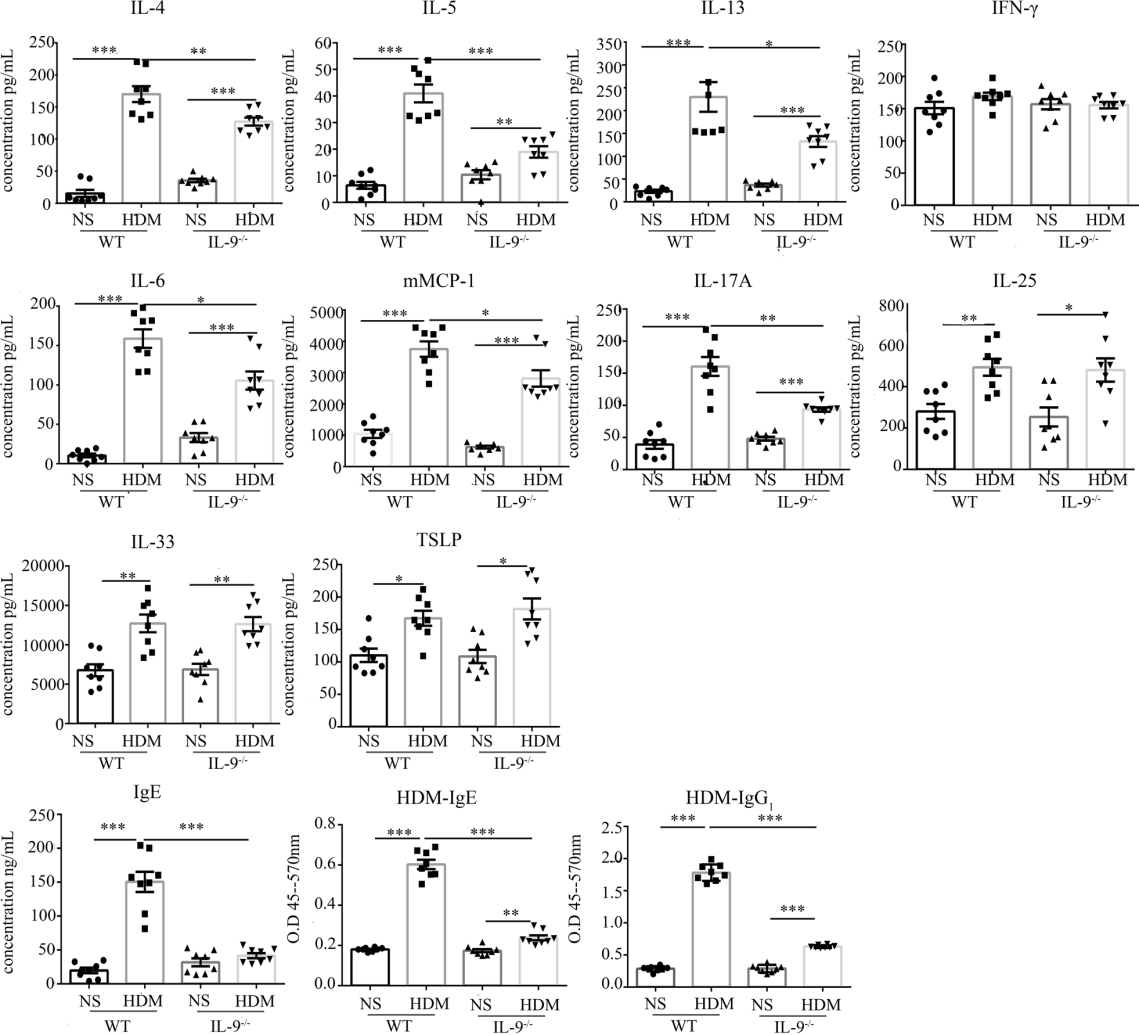
Concentrations of cytokines in lung homogenates as well as serum IgE and IgG_1_ of WT and *Il9*^*−/−*^mice challenged with HDM and NS. Bars show the mean ± SEM (n = 8 in each group). *p < 0.05, **p < 0.01, ***p < 0.001

ELISA analysis of lung homogenates revealed that HDM challenge regimens caused significantly elevated expression of the Th2 cytokines IL-4, IL-5 and IL-13, but not the Th1 cytokine IFN-γ, in WT and *Il9*^*−/−*^ mice compared with saline-challenge (Fig. 4). Intranasal challenge with HDM also induced a marked increase in the concentration of the inflammatory mediators IL-6, mMCP-1 and IL-17A in both WT and *Il9*^*−/−*^mice compared with the relevant saline challenge (Fig. 4). Notably, the concentrations of all of these cytokines were significantly decreased in the *Il9*^*−/−*^ mice compared with the WT mice following HDM challenge (Fig. 4), suggesting that IL-9 deficiency in *Il9*^*−/−*^mice is likely to result in the attenuation of HDM-induced expression of Th2 cytokines. Interestingly, HDM challenge also markedly and significantly elevated the expression of IL-25, IL-33 and TSLP (three cytokines also thought to a play pivotal role in the initiation of asthma) in the lung tissue of both WT and *Il9*^*−/−*^mice compared with saline challenge. However, the expression of these cytokines was not significantly different in the HDM-challenged *Il9*^*−/−*^ mice and WT mice, indicating that IL-9 deficiency is unlikely to influence the expression of these cytokines (Fig. 4). Albeit, HDM challenge induced a marked increase in the concentrations of total serum IgE in WT mice, it cannot alter the concentration of total serum IgE in the *Il9*^*−/−*^mice. Although HDM exposure in both *Il9*^*−/−*^ mice and WT mice can induce an elevated concentration of HDM-specific IgE and IgG_1_ in the sera, the concentrations of HDM-specific IgE and IgG_1_ of serum significantly decreased in the *Il9*^*−/−*^ mice in comparison to the WT mice with HDM challenge. (Fig. 4).

### IL-9 deficiency reduced ILC2 cell, Th2 cell, and mast cell accumulation in the lung tissue of HDM-challenged mice

**Fig. 5:**
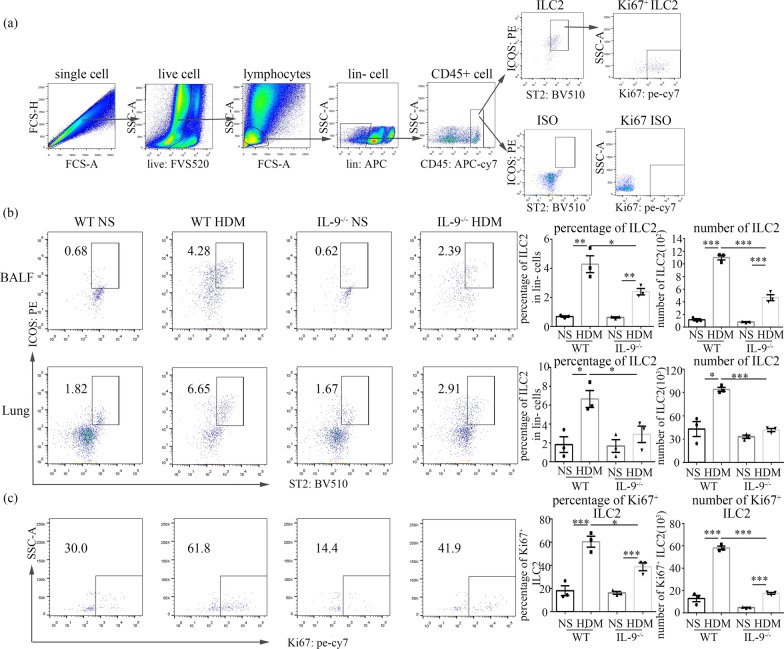
Flow cytometric analysis of ILC2 cells in lung and BALF. **a** The protocol for gating ILC2 cells (Lin^−^CD45^+^ST2^+^ICOS^+^). **b** Flow cytometric identification of ILC2 cells in single-cell suspensions of the BAL fluid (BALF) and lung parenchyma (Lung) of WT and *Il9*^*−/−*^mice challenged with HDM and NS. Left: representative plots of ST2^+^ICOS^+^ ILC2 cells of indicated mice. Right: the percentages of ILC2 cells in lineage-negative cells from BALF and lung. And total number of ILC2 cells in BALF and lung. **c** Flow cytometric analysis of ILC2 cells expressing Ki67 in lung parenchyma. Left: representative plots of Ki67^+^ILC2 cells of indicated mice. Right: the percentages and absolute number of Ki67^+^ILC2 cells in lung parenchyma. Bars show the mean ± SEM (n = 3 in each group). *p < 0.05, **p < 0.01, ***p < 0.001

**Fig. 6:**
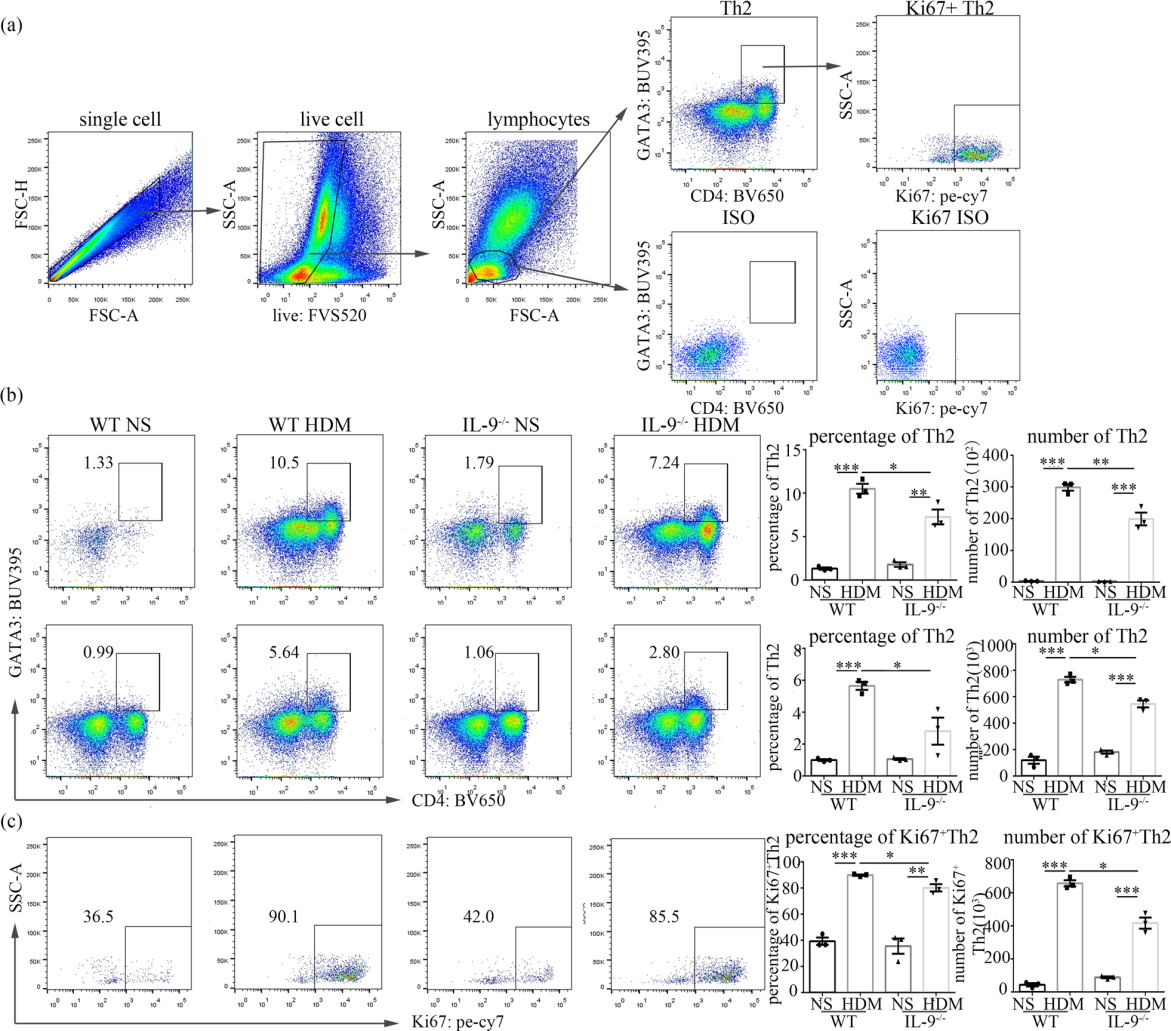
Flow cytometric analysis of Th2 cells in lung and BALF. **a** The protocol for gating Th2 cells (CD4^+^GATA3^+^). **b** Flow cytometric identification of Th2 cells in single-cell suspensions of the BAL fluid (BALF) and lung parenchyma (Lung) of WT and *Il9*^*−/−*^mice challenged with HDM and NS. Left: representative plots of CD4^+^GATA3^+^ Th2 cells of indicated mice. Right: the percentages of Th2 cells in lymphocytes from BALF and lung. And total number of Th2 cells in BALF and lung. **c** Flow cytometric analysis of Th2 cells expressing Ki67 in lung parenchyma. Left: representative plots of Ki67^+^Th2 cells of indicated mice. Right: the percentages and absolute number of Ki67^+^Th2 cells in lung parenchyma. Bars show the mean ± SEM (n = 3 in each group). *p < 0.05, **p < 0.01, ***p < 0.001

**Fig. 7:**
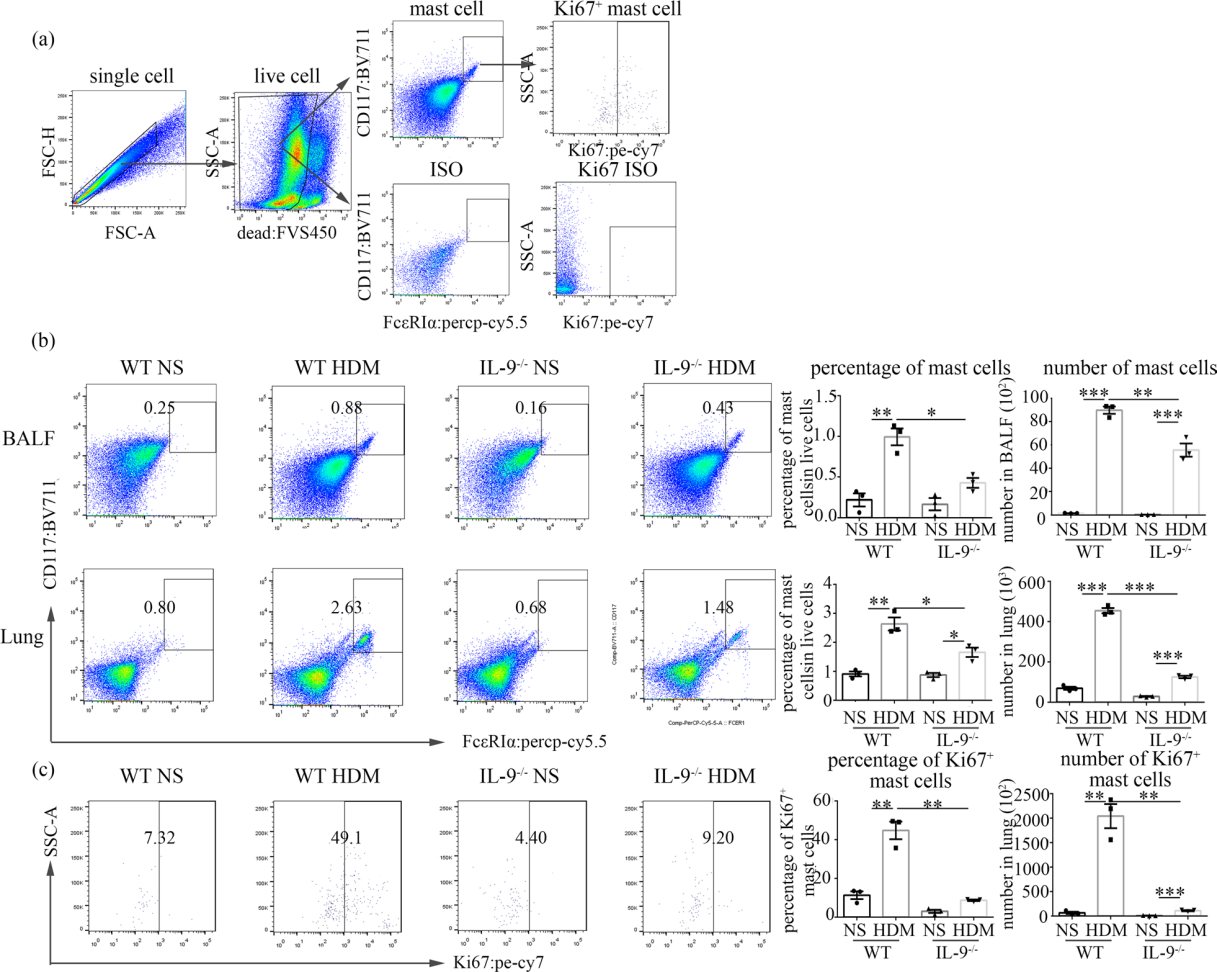
Flow cytometric analysis of mast cells in lung and BALF. **a** The protocol for gating mast cells (CD117^+^ FcεRIα^+^). **b** Flow cytometric identification of mast cells in single-cell suspensions of the BAL fluid (BALF) and lung parenchyma (Lung) of WT and *Il9*^*−/−*^mice challenged with HDM and NS. Left: representative plots of CD117^+^ FcεRIα^+^ mast cells of indicated mice after gating out dead cells. Right: the percentages of mast cells in BALF and lung. And total number of mast cells in BALF and lung. **c** Flow cytometric analysis of mast cells expressing Ki67 in lung parenchyma. Left: representative plots of Ki67^+^mast cells of indicated mice. Right: the percentages and absolute number of Ki67^+^mast cells in lung parenchyma. Bars show the mean ± SEM (n = 3 in each group). *p < 0.05, **p < 0.01, ***p < 0.001

Flow cytometry showed that HDM challenge induced a dramatic increase in the percentages and absolute numbers of ILC2 cells (Fig. 5), Th2 cells (Fig. 6) and mast cells (Fig. 7) in the lung tissues and BALF of both WT and *Il9*^*−/−*^mice compared to the control saline challenge; and the HDM-induced increases in these infiltrating cells were significantly attenuated in the HDM-challenged *Il9*^*−/−*^ mice compared with the HDM challenged WT animals (Figs. 5b, 6b, 7b). The significantly elevated numbers of mast cells in the *Il9*^*−/−*^mice challenged with HDM compared with saline control is consistent with the change in the expression of mMCP-1 (Fig. 4).

### IL-9 deficiency inhibited the proliferation of ILC2, Th2 and mast cells

We assessed expression of Ki67 in ILC2, Th2 and mast cells in the lung tissues. As expected, the percentages and mean number of Ki67^+^ILC2 cells (Fig. 5c), Ki67^+^Th2 cells (Fig. 6c), and Ki67^+^mast cells (Fig. 7c) were significantly increased in the lung tissues of the WT mice with HDM challenge in comparison to the saline control challenge. In contrast, depletion of the IL-9 gene significantly attenuated the percentages and total number of Ki67^+^ILC2 cells (Fig. 5c), Ki67^+^Th2 cells (Fig. 6c), and Ki67^+^ mast cells (Fig. 7c) in the lung tissues of the HDM-challenged *Il9*^*−/−*^mice compared with HDM-challenged WT mice.

## Discussion

IL-9, a pleiotropic cytokine, has been implicated in the pathogenesis of many atopic diseases; including allergic rhinitis, asthma, atopic dermatitis, contact dermatitis, and food allergy; through a variety of activities on mast cells, epithelial cells and smooth muscle cells [18–20]. Previous studies have addressed the role of IL-9 in inducing changes associated with human asthma by investigating the airways of animals by blockade or genetic knock-down of IL-9 designed to induce allergic inflammation or over expression of IL-9 [6, 9, 21]. The present study further explored the role of IL-9 by comparing the differences in airway inflammation and percentages and numbers of target cells (ILC2 cells, Th2 cells and mast cells) in murine asthma models of WT and *Il9*^*−/−*^mice challenged with HDM. It has been well documented that type 2 cytokines IL-5 and IL-13 are implicated in the pathogenesis of the eosinophil-rich airway inflammation, which typically characterizes asthma [22, 23]. In the present study we demonstrated that IL-9 deficiency clearly attenuated the production of type 2 cytokines. This finding is in accordance with a previous study, which showed that IL-9 blockade reduced the concentrations of these mediators in the BALF of OVA-sensitized and challenged mice [8]. Using HDM-challenged murine models of asthma, our data further indicated that IL-9 deficiency reduced proliferation of target cells (ILC2 cells, Th2 cells and mast cells) and infiltration of eosinophils. Furthermore, IL-9 deficiency may also partly account for reduced goblet cell hyperplasis and hypertrophy observed in the present study, particularly as type 2 cytokines and eosinophils have been implicated as contributory factors to these phenomena [24–26].

The epithelium-derived cytokines IL-33, TSLP, and IL-25 have been implicated in pathogenesis of asthma because they promote type 2 cytokine synthesis [27, 28]. Correspondingly, our data showed that the expression of these cytokines increased in the HDM-sensitized and challenged mice. Interestingly, IL-9 deficiency did not further reduce the expression of IL-33, IL-25 and TSLP, although the airway infiltration of inflammatory cells, goblet cell hyperplasia, and type 2 cytokines production were attenuated. Moreover, as shown in our related previous study, per-nasal delivery of IL-33 alone to the airway of wild-type mice was apparently sufficient to induce all of the pathophysiological processes in the airways associated with asthma, including AHR, predominantly eosinophilic, goblet cell hyperplasia. All of these changes were however attenuated in the IL-33-challenged *Il9*^*−/−*^ mice [17]. In addition, overexpression of TSLP in the lung stimulated IL-9 production in vivo, and anti-IL-9 treatment attenuated TSLP-induced airway inflammation [29]. These observations firmly support the hypothesis that IL-9 may be a critical downstream component of IL-33, IL-25 and TSLP signaling pathways in asthma pathogenesis.

It has been reported that ILC2 cells are able to produce IL-9, which is crucial for survival of the ILC2 cells [30]. Whilst the numbers of ILC2s in helminth-induced lung inflammation have been shown to be reduced in IL-9 receptor deficient mice [30], neutralization of IL-9 has not been found to affect the ILC2 numbers in lung tissues in a papain-induced murine model of lung inflammation [21]. However, our data have shown that IL-9 deficiency markedly attenuated ILC2 cell accumulation and production of IL-5 and IL-13, which are consistent with the findings from other studies. For example, IL-9 deficiency in ILC2 cells led to a decrease in IL-5 and IL-13 production in cells cultured in the presence of TSLP and IL-33, despite the number of ILC2 cells not being affected [21, 31, 32]. Taken together, IL-9 can promote the proliferation and survival of ILC2 cells, as well as production of IL-5 and IL-13, suggesting that IL-9 may promote allergic inflammation through amplifying the function of ILC2.

With reference to the different Th subsets, it has been shown that IL-9 acts as a growth factor for especially Th2 and Th17 cells [13, 20]. Previous studies have shown that anti-IL-9 antibody treatment was able to inhibit airway inflammation by reducing the number of Th17 cells and IL-17 levels [9]. Additionally, it has been also shown that IL-9 mediates Th17 differentiation in vitro [33, 34]. Furthermore, pulmonary overexpression of IL-9 appears to induce Th2 differentiation leading to pathologic changes in the lungs [35]. Our findings for Th2 cells are also in accordance with these studies, and have further demonstrated that IL-9 deficiency significantly decreased Th2 cells infiltration and production of IL-4, IL-5, IL-13 and IL-17A; suggest that IL-9 can act on Th2 cells and Th17 cells, which are major contributors to allergic inflammation, either directly or indirectly.

Mast cells are a main target of IL-9, which serves as a growth factor for these cells and also influences their function [36, 37]. The present study demonstrated that mast cell accumulation in the lung tissue of HDM-challenged mice was clearly attenuated by IL-9 deficiency, and this was supported by the reduction of mMCP-1 in the lung tissue homogenate of the experimental animals. These findings are in accordance with previous studies, which indicated that accumulation of mast cells in a model of acute allergic inflammation was dependent on IL-9, because neutralizing IL-9 dramatically reduced the number of mast cells infiltrating the lungs [10, 38]. These data suggest that IL-9 inhibition might be required to diminish pulmonary mast cell numbers, possibly providing some beneficial effects in asthmatics.

Despite of the induction of mucus and Th2 cytokines in OVA-induced acute allergic asthma model, IL-9 slightly affects the cellular changes in asthma [10]. To understand the role of IL-9 in the inflammation process of asthma, the mice in the present study were sensitized by intraperitoneal injection of HDM in aluminum salts to initiate sever lung inflammation with obvious cellular changes. According to previously studies [15, 39], aluminum salts was used as an adjuvant to provide HDM protein antigen sustained release and non-specific immune stimulation. In this point of view, the experimental model in the present study can possibly help us to clearly clarify the role of IL-9 in airway inflammation than that of naturally aerosol inhalation of allergens [40, 41].

Asthma has a higher incidence in females, and female asthmatic patients seem to experience severer asthma symptoms than those of males, resulting in a reduced quality of life [42]. In addition, female mice easily suffer from OVA-induced type 2 dominated asthma-like changes than those of male mice [43]. Furthermore, considering a protective role of IL-9 receptor specific single nucleotide polymorphisms against wheezing in males, we established a murine asthmatic model in female mice [44], without interference the behaviours of male mices y[45]. Still, we can not exclude that the results observed in this study are also applicable to males. More studies are certainly required to figure out this question.

Our studies have shown that IL-9 deficiency markedly reduced the numbers of ILC2 cells, Th2 cells and mast cells, in BALF and the lung tissues of HDM-challenged mice. Thus, it is reasonable to speculate that IL-9 deficiency might inhibit ILC2 cells, Th2 cells and mast cells accumulation in the lung by leading to decreased proliferation of these cells. Indeed, assessment of the expression of Ki67 in these cells, as a marker for cells that were in active phases of the cell cycle, demonstrated that the number of Ki67^+^ILC2 cells, Ki67^+^Th2 cells and Ki67^+^mast cells in the *Il9*^*−/−*^mice was clearly reduced compared to that in WT mice following HDM challenge, thus supporting the hypothesis that IL-9 deficiency may partly affect proliferation of ILC2, Th2 and mast cells.

## Conclusion

In summary, our study has demonstrated a critical role for IL-9 in regulating numbers of ILC2 cells, Th2 cells, mast cells and expression of cytokines produced by these cells in the airways of HDM sensitized mice, following allergen challenge. Furthermore, IL-9 deficiency results in a profound decrease in inflammatory cellular infiltration and goblet cell hyperplasia. It is possible that IL-9 might promote proliferation of ILC2 cells, Th2 cells and mast cells. These findings should improve the understanding of the pathogenesis of asthma and facilitate the development of novel therapies for treatment of this disease.

## Data Availability

Upon request of corresponding author.

## Abbreviations

WT: Wild type
Il9^−^^/^^−^: IL-9-deficient
HDM: House dust mite
NS: Saline
BALF: Bronchoalveolar lavage fluid
AHR: Airways hyperresponsiveness
Rrs: Airways resistance
Mch: Methacholine
H&E: Haematoxylin& eosin
PAS: Periodic acid-Schiff
mMCP-1: Mast cell protease 1
TSLP: Thymic stromal lymphopoietin

## References

[1] Loftus PA, Wise SK (2016). Epidemiology of asthma. Curr Opin Otolaryngol Head Neck Surg.

[2] Boonpiyathad T, Sözener ZC, Satitsuksanoa P, Akdis CA (2019). Immunologic mechanisms in asthma. Semin Immunol.

[3] Gong F, Pan YH, Huang X, Zhu HY, Jiang DL (2017). From bench to bedside: therapeutic potential of interleukin-9 in the treatment of asthma. Exp Therap Med.

[4] Hoppenot D, Malakauskas K, Lavinskienė S, Bajoriūnienė I, Kalinauskaitė V, Sakalauskas R (2015). Peripheral blood Th9 cells and eosinophil apoptosis in asthma patients. Medicina (Kaunas).

[5] Jia L, Wang Y, Li J (2017). Detection of IL-9 producing T cells in the PBMCs of allergic asthmatic patients. BMC Immunol.

[6] Temann UA, Ray P, Flavell RA (2002). Pulmonary overexpression of IL-9 induces Th2 cytokine expression, leading to immune pathology. J Clin Invest.

[7] Temann UA, Laouar Y, Eynon EE, Homer R, Flavell RA (2007). IL9 leads to airway inflammation by inducing IL13 expression in airway epithelial cells. Int Immunol.

[8] Kim MS, Cho KA, Cho YJ, Woo SY (2013). Effects of interleukin-9 blockade on chronic airway inflammation in murine asthma models. Allergy Asthma Immunol Res.

[9] Shin JH, Kim DH, Kim BY (2017). Anti-interleukin-9 antibody increases the effect of allergen-specific immunotherapy in murine allergic rhinitis. Allergy Asthma Immunol Res.

[10] Kearley J, Erjefalt JS, Andersson C (2011). IL-9 governs allergen-induced mast cell numbers in the lung and chronic remodeling of the airways. Am J Respir Crit Care Med.

[11] Cheng G, Arima M, Honda K (2002). Anti-interleukin-9 antibody treatment inhibits airway inflammation and hyperreactivity in mouse asthma model. Am J Respir Crit Care Med.

[12] Matsuzawa S, Sakashita K, Kinoshita T, Ito S, Yamashita T, Koike K (2003). IL-9 enhances the growth of human mast cell progenitors under stimulation with stem cell factor. J Immunol.

[13] Chakraborty S, Kubatzky KF, Mitra DK (2019). An update on interleukin-9: from its cellular source and signal transduction to its role in immunopathogenesis. Int J Mol Sci.

[14] Gu ZW, Wang YX, Cao ZW (2017). Neutralization of interleukin-9 ameliorates symptoms of allergic rhinitis by reducing Th2, Th9, and Th17 responses and increasing the Treg response in a murine model. Oncotarget.

[15] Li Y, Chen S, Chi Y (2019). Kinetics of the accumulation of group 2 innate lymphoid cells in IL-33-induced and IL-25-induced murine models of asthma: a potential role for the chemokine CXCL16. Cell Mol Immunol.

[16] An G, Wang W, Zhang X (2020). Combined blockade of IL-25, IL-33 and TSLP mediates amplified inhibition of airway inflammation and remodelling in a murine model of asthma. Respirology.

[17] Du X, Li C, Wang W (2020). IL-33 induced airways inflammation is partially dependent on IL-9. Cell Immunol.

[18] Townsend JM, Fallon GP, Matthews JD, Smith P, Jolin EH, McKenzie NA (2000). IL-9-deficient mice establish fundamental roles for IL-9 in pulmonary mastocytosis and goblet cell hyperplasia but not T cell development. Immunity.

[19] Angkasekwinai P (2019). Th9 cells in allergic disease. Curr Allergy Asthma Rep.

[20] Koch S, Sopel N, Finotto S (2017). Th9 and other IL-9-producing cells in allergic asthma. Semin Immunopathol.

[21] Wilhelm C, Hirota K, Stieglitz B (2011). An IL-9 fate reporter demonstrates the induction of an innate IL-9 response in lung inflammation. Nat Immunol.

[22] Huang P, Li Y, Lv Z (2017). Comprehensive attenuation of IL-25-induced airway hyperresponsiveness, inflammation and remodelling by the PI3K inhibitor LY294002. Respirology.

[23] Lou H, Zhang N, Bachert C, Zhang L (2018). Highlights of eosinophilic chronic rhinosinusitis with nasal polyps in definition, prognosis, and advancement. Int Forum Allergy Rhinol.

[24] Ito R, Maruoka S, Soda K (2018). A humanized mouse model to study asthmatic airway inflammation via the human IL-33/IL-13 axis. JCI Insight.

[25] Kanoh S, Tanabe T, Rubin BK (2011). IL-13-induced MUC5AC production and goblet cell differentiation is steroid resistant in human airway cells. Clin Exp Allergy.

[26] Zhao J, Minami Y, Etling E (2017). Preferential Generation of 15-HETE-PE Induced by IL-13 regulates goblet cell differentiation in human airway epithelial cells. Am J Respir Cell Mol Biol.

[27] Akdis CA, Arkwright PD, Brüggen MC (2020). Type 2 immunity in the skin and lungs. Allergy.

[28] Lan F, Zhang N, Holtappels G (2018). Staphylococcus aureus induces a mucosal type 2 immune response via epithelial cell-derived cytokines. Am J Respir Crit Care Med.

[29] Yao W, Zhang Y, Jabeen R (2013). Interleukin-9 is required for allergic airway inflammation mediated by the cytokine TSLP. Immunity.

[30] Turner JE, Morrison PJ, Wilhelm C (2013). IL-9-mediated survival of type 2 innate lymphoid cells promotes damage control in helminth-induced lung inflammation. J Exp Med.

[31] Mohapatra A, Van Dyken SJ, Schneider C, Nussbaum JC, Liang HE, Locksley RM (2016). Group 2 innate lymphoid cells utilize the IRF4-IL-9 module to coordinate epithelial cell maintenance of lung homeostasis. Mucosal Immunol.

[32] Rauber S, Luber M, Weber S (2017). Resolution of inflammation by interleukin-9-producing type 2 innate lymphoid cells. Nat Med.

[33] Chowdhury K, Kumar U, Das S (2018). Synovial IL-9 facilitates neutrophil survival, function and differentiation of Th17 cells in rheumatoid arthritis. Arthritis Res Ther.

[34] Li H, Nourbakhsh B, Cullimore M, Zhang GX, Rostami A (2011). IL-9 is important for T-cell activation and differentiation in autoimmune inflammation of the central nervous system. Eur J Immunol.

[35] de Heusch M, Steenwinckel V, Cochez PM (2020). IL-9 exerts biological function on antigen-experienced murine T cells and exacerbates colitis induced by adoptive transfer. Eur J Immunol.

[36] Steenwinckel V, Louahed J, Lemaire MM (2009). IL-9 promotes IL-13-dependent paneth cell hyperplasia and up-regulation of innate immunity mediators in intestinal mucosa. J Immunol.

[37] Meiners J, Reitz M, Rüdiger N (2020). IL-33 facilitates rapid expulsion of the parasitic nematode Strongyloides ratti from the intestine via ILC2- and IL-9-driven mast cell activation. PLoS Pathog.

[38] Sehra S, Yao W, Nguyen ET (2015). TH9 cells are required for tissue mast cell accumulation during allergic inflammation. J Allergy Clin Immunol.

[39] Chen S, Han Y, Chen H (2018). Bcl11b regulates IL-17 through the TGF-β/Smad pathway in HDM- induced asthma. Allergy Asthma Immunol Res.

[40] Ren M, Feng M, Long Z (2021). Allergic asthma-induced cognitive impairment is alleviated by dexamethasone. Front Pharmacol.

[41] Sun Z, Ji N, Ma Q (2020). Epithelial-mesenchymal transition in asthma airway remodeling is regulated by the IL-33/CD146 axis. Front Immunol.

[42] Chowdhury NU, Guntur VP, Newcomb DC (2021). Sex and gender in asthma. Eur Respir Rev.

[43] Okuyama K, Wada K, Chihara J (2008). Sex-related splenocyte function in a murine model of allergic asthma. Clin Exp Allergy.

[44] Melén E, Gullstén H, Zucchelli M (2004). Sex specific protective effects of interleukin-9 receptor haplotypes on childhood wheezing and sensitisation. J Med Genet.

[45] Meeh KL, Rickel CT, Sansano AJ (2021). The development of sex differences in the nervous system and behavior of flies, worms, and rodents. Dev Biol.

